# Hydration sites of unpaired RNA bases: a statistical analysis of the PDB structures

**DOI:** 10.1186/1472-6807-11-41

**Published:** 2011-10-19

**Authors:** Svetlana Kirillova, Oliviero Carugo

**Affiliations:** 1 Department of Structural and Computational Biology, Max F. Perutz Laboratories, Vienna University, Campus Vienna Biocenter 5, A-1030 Vienna, Austria; 2 Department of Chemistry, Pavia University, Viele Taramelli 12, I-27100 Pavia, Italy

## Abstract

**Background:**

Hydration is crucial for RNA structure and function. X-ray crystallography is the most commonly used method to determine RNA structures and hydration and, therefore, statistical surveys are based on crystallographic results, the number of which is quickly increasing.

**Results:**

A statistical analysis of the water molecule distribution in high-resolution X-ray structures of unpaired RNA nucleotides showed that: different bases have the same penchant to be surrounded by water molecules; clusters of water molecules indicate possible hydration sites, which, in some cases, match those of the major and minor grooves of RNA and DNA double helices; complex hydrogen bond networks characterize the solvation of the nucleotides, resulting in a significant rigidity of the base and its surrounding water molecules. Interestingly, the hydration sites around unpaired RNA bases do not match, in general, the positions that are occupied by the second nucleotide when the base-pair is formed.

**Conclusions:**

The hydration sites around unpaired RNA bases were found. They do not replicate the atom positions of complementary bases in the Watson-Crick pairs.

## Background

Water plays an important role in the function of biological molecules [1]. In the case of RNA, water is regarded as "an integral part of nucleic acid structure" because it defines structure and folding and participates in intra-molecular interactions [2]. For example, the free energy of RNA hairpins is considerably influenced by the presence of acceptor groups that do not form hydrogen bonds [3]. Moreover, RNA interactions with other molecules can be mediated by water molecules that link the two partners and water molecules bound to RNA can be replaced by polar groups of other molecules [4,5]. Furthermore, water molecules having large dipole moments modulate electrostatic interactions [6]. For these reasons, the understanding of RNA hydration is of crucial importance in drug design and modeling interactions [7]. Additionally, molecular dynamics simulations require the definition of the initial positions of the solvent atoms and the last stages of crystallographic refinements involve RNA hydration sites [8].

The most frequently used experimental methods to study RNA hydration are X-ray crystallography and NMR spectroscopy. NMR spectroscopy gives "dynamic viewpoints" of the micro-heterogeneity of the local hydration structure and provides information about the hydration kinetics [9-13]. Crystallographic results are on the contrary considered, in general, as "static views", though some information about solvent dynamics can be obtained from the atomic displacement parameters, known also as B-factors [14,15]. Additionally, the positions of the hydrogen atoms are usually not determined by crystallography and, as a consequence, the detailed description of the hydrogen bond stereochemistry is impossible. Nevertheless, X-ray crystallography remains the most commonly used method to determine RNA structures and hydration and, therefore, statistical surveys must be based on crystallographic results, the number of which is quickly increasing.

The earliest statistical analyses were focused on DNA molecules [16,17]. Hydration sites were revealed with three-dimensional solvent density maps, built on the basis of 40 high-resolution X-ray DNA structures, and "hydration building blocks" were made for every particular base type [16,17]. Auffinger and Westhof extended this approach to RNA molecules [15,18] and a web server (SwS) was developed [19]. It provides the solvation of DNA, RNA, and hybrid base pairs by using the generation of 3D solvent density maps. However, most of the RNA structures are rather irregular and contain many unpaired nucleotides [20,21], which, as a consequence, are not analyzed by the SwS web-server.

In the present study, the high-resolution X-ray structures of unpaired RNA bases were analyzed. By using Monte Carlo simulations and cluster analysis methods, we found hydration sites around unpaired RNA bases, which appear to be tightly bound to the nucleotides. Many of these solvation sites, which participate in complex hydrogen bond networks, match the hydration sites found in the major and minor grooves of RNA and DNA double helices.

## Results and Discussion

### Is the extent of hydration around the bases different?

The method described in reference [17], which is based on the statistical *zI *test [22], was used to compare the extent of hydration around the two pyrimidine and the two purine bases. For example, given a dataset with *O_x_*, the number of oxygen atoms around cytosine bases; *O_y_*, the number of oxygen atoms around uracil bases; *F_x_*, the number of cytosine bases; and *F_y_*, the number of uracil bases, it is possible to compute the expected number of water molecules (*E_x_*) around cytosines

(1) $$E_{x}=\frac{F_{x}\left(O_{x}+O_{y}\right)}{F_{x}+F_{y}}.$$

The fraction of uracil bases in the dataset is

(2) $$P_{y}=\frac{F_{y}}{F_{x}+F_{y}},$$

and the value of *z *for cytosines can be computed as

(3) $$z=\frac{\left(|E_{x}-O_{x}|-c\right)}{\sqrt{P_{y}E_{x}}},$$

where *c *is equal to 0.2 if *E_x _*>*O_x _*or to 0.5 if *E_x _*<*O_x_*. For example, for cytosine versus uracil, the following values can be seen in Table 1: *O_x_*. = 250, *O_y _*= 373, *F_x _*= 82, and *F_y _*= 138; as a consequence, *E_x _*= 232.2, *P_y _*= 0.63, and *c *= 0.5; and therefore *z *= 1.43. The *z *values are then equal to 1.43 for cytosine, 1.46 for uracil, 0.23 for adenine and to 0.22 for guanine. Given that the *z *threshold values are 1.64, at the 10% probability level, and 1.96, at the 5% level of probability, it can be concluded the hydration extent of pyrimidine and purine bases is the statistically the same. The smaller unpaired cytosines and uracils, on the one hand, and the larger unpaired adenines and guanines, on the other, have the same penchant to be surrounded by water molecules.

**Table 1:** Crystallographic data used to study the hydration of the unpaired RNA bases

Base	Total number of bases	Number of bases*	Number of unpaired bases*	Number of waters around unpaired bases*
Adenine	18.756	405	197	719

Guanine	25.286	554	128	474

Cytosine	19.548	407	82	250

Uracil	14.032	355	138	373

* Data obtained for the X-ray RNA structures with the resolution better than 2.0 Å

### Clustering tendency

The clustering tendency of the water molecules around the unpaired RNA bases was estimated by means of the well-known Hopkins statistics [23], which compares two alternative hypotheses: uniform distribution of water molecules, on the one hand, and natural organization into discrete clusters, on the other.

Let *X *= {*x_i_*, *i *= l to *n*} to be a collection of *n *observed water molecules and let *Y *= {*y_i_*, *i *= l to *m*} to be an ensemble of *m *random points within the space occupied by the water molecules. Let be *m *< <*n*. Two types of distances are defined: *u_j _*is the minimal distance between *y_j _*and its nearest element of *X; *and *w_j _*is the minimal distance from the randomly selected j^th ^element of *X *to its nearest neighbor element of *X*. The Hopkins statistic is defined as

(4) $$H=\frac{\overset{m}{\underset{j=1}{\sum }}u_{j}}{\overset{m}{\underset{j=1}{\sum }}u_{j}+\overset{m}{\underset{j=1}{\sum }}w_{j}}$$

In present study, *n *is equal to the number of water molecules around the RNA bases (see Table 1); *m *is equal to 20, a number much smaller than the numbers of water molecules located around each type of base. The values of the *H *index range from 0.72, for uracil, to 0.74, for cytosine and guanine, and to 0.78, for adenine. They are considerably higher than the value of 0.5, which is expected for a totally uniform distribution. They are also higher than the value of 0.7, which is usually taken as a threshold, over which the data present a natural tendency to be organized into discrete clusters [23]. Consequently, it can be concluded that the water molecules that surround unpaired RNA bases tend to be located in discrete hydration sites.

### Hydration clusters

Several clusters of water molecules were detected around unpaired RNA bases, only three around cytosine and up to seven around guanine (see Table 2). The coordinates of the water clusters around the unpaired RNA bases are represented in the Additional files: guanine (Additional file 1), adenine (Additional file 2), cytosine (Additional file 3), uracil (Additional file 4). About 30% of the water molecules can be grouped into separate clusters around purine bases (28% for adenine and 32% for guanine) and about 20% around pyrimidine bases (17% for uracil and 18% for cytosine). This implies that many solvent molecules are not found into well-defined hydration sites but are, on the contrary, spread around the bases. This might depend on the fact that the crystal packing and the local environment can be extremely variable from one structure to the next. However, several solvation sites are observable.

**Table 2:** Clusters of water molecules around the unpaired RNA bases

Base	Cluster	Number of waters	Compact-ness, Å	**Distance to the nearest base atom**, Å	**Angle**, degrees	**Torsion**, degrees	Non-base atoms creating H-bonds with the cluster
Adenine	W1	42	1.3	N7-W1 2.6	C5-N7-W1 142	C6-C5-N7-W1 5	OP1, OP2

Adenine	W2	43	0.9	N6-W2 3.4	C6-N6-W2 161	N1-C6-N6-W2 -77	isolated

Adenine	W3	66	1.3	N3-W3 2.8	C4-N3-W3 132	N9-C4-N3-W3 11	W5, O2'

Adenine	W4	25	1.4	N3-W4 (4.6)*	C4-N3-W4 93	N9-C4-N3-W4 -40	O2', O3'

Adenine	W5	25	1.2	N3-W5 (4.7)*	C4-N3-W5 156	N9-C4-N3-W5 60	W3

Guanine	W1	26	1.3	N7-W1 2.7	C5-N7-W1 135	C6-C5-N7-W1 -17	W2

Guanine	W2	17	1.1	O6-W2 2.7	C6-O6-W2 131	N1-C6-O6-W2 170	W1

Guanine	W3	17	1.0	N3-W3 3.1	C4-N3-W3 126	N9-C4-N3-W3 -6	W5, O2'

Guanine	W4	23	0.7	N3-W4 3.1	C4-N3-W4 114	N9-C4-N3-W4 50	O4'

Guanine	W5	5	0.2	N2-W5 3.0	C2-N2-W5 119	N3-C2-N2-W5 -8	W3

Guanine	W6	20	0.6	N2-W6 3.9	C2-N2-W6 123	N3-C2-N2-W6 76	isolated

Guanine	W7	44	1.0	O6-W7 (4.4)*	C6-O6-W7 162	N1-C6-O6-W7 -4	isolated

Uracil	W1	17	1.1	O4-W1 2.6	C4-O4-W1 127	C5-C4-O4-W1 -8	isolated

Uracil	W2	14	1.0	C5-W2 3.7	C6-C5-W2 76	N1-C6-C5-W2 -101	OP2

Uracil	W3	14	0.9	O2-W3 3.5	C2-O2-W3 132	N3-C2-O2-W3 136	O2'

Uracil	W4	12	0.8	N3-W4 2.7	C4-N3-W4 122	C5-C4-N3-W4 170	isolated

Cytosine	W1	24	0.9	N4-W1 2.9	C4-N4-W1 116	C5-C4-N4-W1 2	W3

Cytosine	W2	10	0.9	C5-W2 4.0	C6-C5-W2 85	N1-C6-C5-W2 -127	W3, OP2

Cytosine	W3	12	0.7	C5-W3 3.6	C6-C5-W3 112	N1-C6-C5-W3 -177	W1, W2, OP2

Each cluster is associated with a name (for example W1); the number of water molecules that it contains; the cluster compactness, defined at the maximal distance of a water molecule from the cluster center; the atom of the base that is the closest to it; the distance from this atom; the angle centered on this atom; the torsional angle that monitors deviation of the cluster from the base plane; and the other atoms that might interact with it.

* Distances that are longer than 4.0 Å are placed in parentheses.

**Figure 1:**
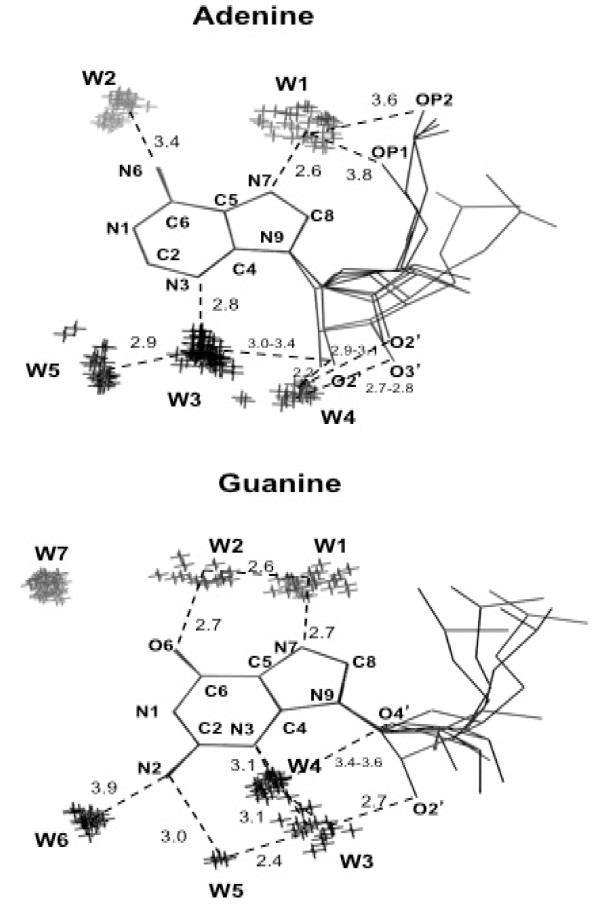
**Possible hydration sites around RNA unpaired purines**. Oxygen atoms of water molecules are represented by crosses (X). All the nucleotide conformations, which were observed more than 15 times, are shown. Hydrogen bonds between hydrophilic groups of nucleotides and hydration sites are represented by dashed lines. Their lengths are indicated in Ångstroms. Figure was prepared using PyMOL [30].

**Figure 2:**
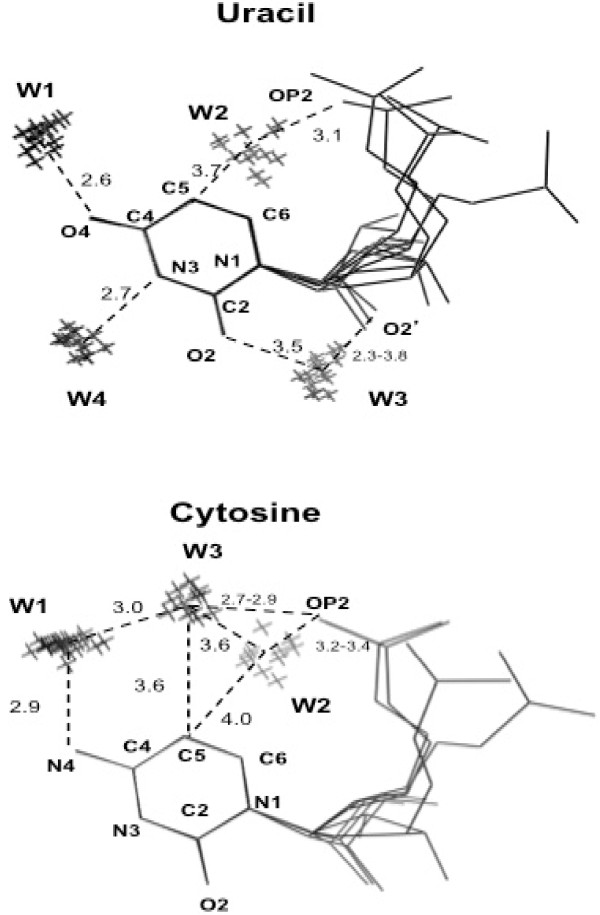
**Possible hydration sites around RNA unpaired pyrimidines**. Oxygen atoms of water molecules are represented by crosses (X). All the nucleotide conformations, which were observed more than 15 times, are shown. Hydrogen bonds between hydrophilic groups of nucleotides and hydration sites are represented by dashed lines. Their lengths are indicated in Ångstroms. Figure was prepared using PyMOL [30].

The Watson-Crick edge contains the atoms involved in the strongest interactions that stabilize the base-pairing. The hydration centers around the unpaired bases do not replicate the positions of the complementary base atoms (Figures 1 and 2). Only one hydration center (W4), located in the area along of the Watson-Crick edge, was detected for the unpaired uracil bases (Figure 2). The Watson-Crick edge of the unpaired bases interacts more frequently with proteins, ligands, and ions than with water molecules (see section "Other non-bonding interactions around unpaired RNA bases").

The dimensions of the hydration clusters are relatively variables (Table 2). Some of them contain several tens of water molecules, like, for example, cluster W3 around adenine (66 molecules) and others are considerably smaller, like cluster W5 around guanine (5 molecules). The compactness of each cluster, measured as the maximal distance of a water molecule from the center of the cluster, ranges from 0.2 Å to 1.4 Å (Table 2).

### Conformational analysis

Since the base hydration may depend on the nucleotide conformation [15], the RNA stereochemistry was examined. It is described by the sugar ring conformation, the mutual arrangement of the sugar and of the base rings, and by six backbone torsion angles [24]. Four main sugar ring puckerings are possible, as it is summarized by the sugar pucker circle reported in the server [24]. Two main arrangements, *anti *and *syn*, are possible for the base and the sugar rings. The backbone torsions tend to have values near 180°, 60°, and -60°, which are called *trans*, *gauche+*, and *gauche-*.

Most of the possible conformations were observed in the structures of free and hydrated nucleotides examined in the present paper. Those that were observed more frequently and that were associated with at least 15 water molecules are listed in Table 3. The atomic coordinates of the conformations of the unpaired base are represented in the Additional files: guanine (Additional file 1), adenine (Additional file 2), cytosine (Additional file 3), uracil (Additional file 4). All of them have *anti *arrangement of the base and of the sugar rings, whereas the stereochemistry of the nucleotide backbone varies considerably. The sugar ring adopts the C3'-endo or the C2'-endo conformation in most of the cases and only one exo conformation is observed for adenine.

**Table 3:** Conformations of the unpaired RNA nucleotide examined in the present paper and corresponding hydration of the bases

Base	Nucleotide conformations containing more than 15 surrounding waters	Number of nucleotides	Number of surrounding waters
Adenine	C3'-endo_anti_-g_t_g_g_t_-g	56	273

Adenine	C3'-endo_anti_t_t_t_g_t_-g	7	77

Adenine	C3'-endo_anti_t_t_g_g_t_-g	7	27

Adenine	C2'-exo_anti_-g_t_g_g_t_-g	6	20

Adenine	C3'-endo_anti_-g_t_t_g_t_-g	2	20

Guanine	C3'-endo_anti_-g_t_g_g_t_-g	43	193

Guanine	C2'-endo_anti_-g_t_g_t_-g_g	12	29

Guanine	C2'-endo_anti_-g_t_g_t_-g_t	3	18

Guanine	C3'-endo_anti_-g_t_g_g_-g_-g	7	17

Uracil	C3'-endo_anti_-g_t_g_g_t_-g	10	55

Uracil	C2'-endo_anti_g_t_g_t_-g_-g	7	34

Uracil	C3'-endo_anti_t_t_t_g_t_-g	4	29

Uracil	C2'-endo_anti_-g_t_g_t_-g_g	4	18

Uracil	C3'-endo_anti_t_t_g_g_t_-g	4	17

Cytosine	C3'-endo_anti_-g_t_g_g_t_-g	20	114

Cytosine	C2'-endo_anti_-g_t_g_t_-g_-g	3	17

Cytosine	C3'-endo_anti_t_t_t_g_t_g	2	17

Cytosine	C3'-endo_anti_-g_t_g_g_t_g	4	16

The conformation *C3'-endo_anti_-g_t_g_g_t_-g *is the most frequently observed, like for the RNA base-pairs [24].

Different conformations exhibit dissimilar degrees of hydration. While the common *C3'-endo_anti_-g_t_g_g_t_-g *conformation has about 5 water molecules around the base, other, less common conformations present larger numbers of water molecules. For example, there are on average 10 water molecules around adenine in the *C3'-endo_anti_t_t_t_g_t_-g *or in the *C3'-endo_anti_-g_t_t_g_t_-g *conformation. However, there are also cases where the degree of hydration is considerable smaller. For example, there are only 2 water molecules, on average, around the guanine in conformation *C3'-endo_anti_t_t_t_g_t_-g *or *C3'-endo_anti_-g_t_g_g_-g_-g*.

The physicochemical reason of this variability is unclear, on the basis of the data presented here. This might depend on the paucity of the data or on the effects of the crystal packing.

### Hydrogen bond network

Hydrogen bonds can be classified according to the interacting atoms or with numerical criteria based on energy or on geometry [25]. In present study, we use the geometric criteria described in [25]: the hydrogen bond is very strong if the distance between donor and acceptor atoms is in the range 2.2-2.5 Å, strong if it is in the range 2.5-3.2 Å, and weak if it is in the range 3.2-4.0 Å.

Pertinent details about the hydrogen bonds around free RNA bases are shown in Table 2. Each cluster of water molecules, represented by its center of mass, is associated with the following variables: the nearest RNA base atom, the distance from the nearest RNA base atom, the bond angle centered on the nearest RNA base atom, and the torsion that monitors the out-of-base-plane distortion of the hydrogen bond. The atoms that define the bond angles and torsions are specified in Table 2. The hydrogen bond networks are also depicted in the Figures 1 and 2.

### Guanine and Adenine

In the present paragraph and in the next one, we describe in detail each hydration clusters observed around unpaired bases, its interactions with the RNA molecules, and we compare it with similar hydration sites observed in other studies of nucleic acid hydration.

The cluster W1, facing the N7 nitrogen atom of guanine and adenine (Figure 1), is compatible to the major groove hydration site that was well detected in RNA and DNA [15-18,26-28]. The N7...W1 interaction was considered to be a strong hydrogen bond, given its distance in the range 2.5-3.1 Å. W1 is located roughly in the base plane and may interact also with the backbone OP1 and OP2 oxygen atoms.

Also the cluster W2, facing the O6 oxygen atom of guanine (Figure 1) or the N6 nitrogen atom of adenine (Figure 1), is compatible to the major groove hydration site found in RNA and DNA double helices [15-18,26-28]. A previous statistical analysis of RNA base-pairs hydration revealed that the cluster W2 is less compact than cluster W1 for guanine [18]. However, in the present analysis of unpaired guanine bases, cluster W1 seems to be slightly less compact than cluster W2 (see Table 2). Furthermore, while cluster W2 lies roughly in the guanine plane and makes a strong hydrogen bond with cluster W1, it is markedly out of the adenine plane and does not interact with cluster W1 of adenine. Another difference between adenine and guanine is the distance of cluster W2 from the base. It is larger for adenine (N6...W2 = 3.4 Å) than for guanine (O6...W2 = 2.7 Å), though this might be explained by the different nature of the HOH...O = and of the H_2_O...HN- hydrogen bonds [17].

The cluster W3 is hydrogen bonded to the N3 nitrogen atom and it lies on the base plane of both adenine and guanine (Table 2 and Figure 1). Such a hydration site was found also in the minor groove of the RNA and DNA helices [15-18,26-28]. The presence of the 2'-hydroxyl group in RNA is a reason of the profound structural and dynamical differences between DNA and RNA molecules. In the detailed study of the high resolution crystal structure of the RNA duplex [r(CCCCGGGG]_2_, an extensive hydration of the 2'-hydroxyl was observed. It consists of four water clusters, representing an approximate tetrahedron around the O2' oxygen atom [27]. The cluster W3 of both adenine and guanine corresponds to one of the tetrahedron vertices, which bridges the N3 nitrogen atom and the 2'-hydroxyl oxygen atom.

Some differences between adenine and guanine appear in cluster W4. In both cases, this cluster of water molecules is connected to the ribose ring. However, the interaction between W4 and the ribose is different: with the O2' and O3' oxygen atoms of adenine and with the O4' oxygen atom of guanine. Moreover, W4 interacts directly with the N3 nitrogen atom of guanine, while the analogous interaction with adenine is extremely weak (if any).

The most important steric difference between adenine and guanine is the N2 nitrogen atom of guanine. This also differentiates the RNA and DNA hydration in the minor groove [15,17,18,26]. A statistical study of RNA X-ray structures revealed significant displacements of the positions of the hydration site facing the N2 nitrogen atom of guanine in the Watson-Crick G-C and in the wobble G-U base pairs [18]. In an analogous study of the DNA base pairs, a well-localized hydration site around the N2 nitrogen atom was not observed [17].

In the present analysis of unpaired RNA guanines, we observed two clusters close to the N2 nitrogen atom, W5 and W6. The cluster W5 lies in the guanine plane and is closer to the atom N2 than the cluster W6, which does not lie in the guanine plane. Cluster W5 is very small (only five water molecules) and compact (see Table 2) and makes a strong hydrogen bond with cluster W3. For adenine, on the contrary, only one cluster of water molecules is observed in this region. It does not interact directly with adenine but it makes a strong hydrogen bond with cluster W3.

Intriguingly, a seventh cluster of water molecules is observed around guanine (cluster W7; see Table 2 and Figure 1). It is one of the largest (44 water molecules) and relatively compact. However, this cluster is isolated (more than 4 Å) from any nucleotide atoms and any water molecule of other clusters and it does not participate in the hydrogen bond network around unpaired guanine bases.

### Cytosine and Uracil

Around both cytosine and uracil there is a cluster of water molecules (W1, Figure 2) that is hydrogen bonded to the hydrophilic group in position 4, the nitrogen atom N4 of cytosine and the oxygen atom O4 of uracil. This interaction seems to be quite strong given the short inter-atomic distance and the fact that the water molecules tend to lie on the base plane (Table 2). A similar, strong hydrogen bond was observed in the major groove of RNA and DNA double helices [15-18,26-28].

A second cluster of water molecules (W2, Figure 2) is also observed for both cytosine and uracil. It bridges the base and the phosphate, by means of a week hydrogen bond with the CH group of the base [25] and a strong or weak hydrogen bond with the OP2 oxygen atom (Table 2). Despite a position that it is markedly out of the plane of the base (Table 2), it was observed also in statistical analyses of X-ray structures of RNA base pairs [18].

A third cluster (W3, Figure 2) is observed only around cytosine. It is part of the base-phosphate N4- O_w_- O_w _-OP2 bridge that contains two water molecules and that is well known and characteristic of nucleic acids [15]. The structural element W2-OP2-W3 around the cytosine (Figure 2), with an angle of about 75 degrees, was found also in the high resolution (1.25 Å) A-DNA crystal structure of d(CCCGACGG) [26].

The weak C-H...O hydrogen bonds are important in the structure of biological molecules [25,29]. In the present study, such interactions were detected for the pyrimidines. One cluster of uracil (W2; Figure 2) and two clusters of cytosine (W2, W3; Figure 2) make weak hydrogen bonds with the C5 carbon atom (Table 2). In a statistical analysis of the crystallographic RNA structures [29], it was shown that the C5 carbon atom of the pyrimidines is involved in hydrogen bonds more often than other carbon atoms and that the interactions between water molecules and the C5 carbon atom are stronger in cytosine than in uracil [29].

No other clusters of water molecules were observed around cytosine. On the contrary, two other clusters are present around uracil. One of them (W3, Figure 2) bridges the O2 oxygen atom of the base and the O2' hydroxyl oxygen atom of the ribose, being severely distorted from the uracil plane. It was also observed in the minor groove of uracils [15,18,26]. The other (W4, Figure 2) lies in the base plane and is strongly hydrogen bonded to the N3 nitrogen atom of uracil. Interestingly, this is the only cluster of water molecules that occupies the place that is taken by another RNA base atom (the N1 nitrogen atom of adenine) when a base pair (the Watson-Crick A-U) is formed.

### Other non-bonding interactions around unpaired RNA bases

Only for uracil, all the hydrophilic atoms of the base were found to be hydrogen-bonded to a cluster of water molecules. The N1 atom of both purines and the N3 and O2 atoms of cytosine were found to lack a well clustered hydration site (Figures 1 and 2).

Therefore, we studied the crystal packing contacts in the X-ray structures to verify if these potential hydration sites might be occupied by symmetry related atoms. The "symexp" command of the PyMOL program [30] was used to generate crystal symmetry-related atoms within 5 Å around the reference molecules of the pdb files. Furthermore, we examined the interactions (within 5 Å) with other RNA nucleotides within the same asymmetric unit, which cannot be considered a proper pairing of two nucleotides, and also with proteins, ligands, and ions.

The numbers of the atom-atom interactions, involving the unpaired RNA bases, are shown in Table 4. Less than 10% of the unpaired RNA bases participate in the interactions with another RNA nucleotides within the same asymmetric unit. 12-15% of the unpaired adenine, guanine and cytosine bases are involved in crystal packing contacts. The fraction of uracils involved in crystal packing is slightly larger (22%). The frequency of interactions with atoms of proteins, ligands and ions is rather variable: 40% of cytosines, 44% of adenines, 54% of uracils, and 73% of guanines are in contact with these type of molecules. Most of these interactions were found in the large ribosome complexes.

**Table 4:** Other non-bonding interactions around unpaired RNA bases

	Interactions with RNA atoms			Crystal packing interactions			Interactions with atoms of proteins, ligands, and ions		
**Base**	N_b_*	N_a_**	***d***	N_b_*	N_a_**	***d***	N_b_*	N_a_**	***d***

Adenine	7	92	5	25	325	12	87	1.868	55

Guanine	7	93	5	20	258	11	96	2.381	68

Cytosine	7	41	-	10	98	5	33	532	19

Uracil	5	30	-	30	351	13	75	1.071	34

Number of the unpaired RNA bases participating in the atom-atom interactions within 5 Å and number of atoms around these unpaired bases. For example, 25 from 197 unpaired adenine bases (see the numbers of the unpaired bases in Table 1) have crystal packing contacts within 5 Å and the number of surrounding atoms that participate in these interactions is 325. The empirical parameter *d*, used for cluster detection (see Method section), is also reported for each type of interaction.

* - number of unpaired bases.

** - number of atoms surrounding the base

The same clustering procedure used to identify hydration sites was employed to analyze the spatial distribution of all the atoms that surround the unpaired RNA bases. The values of the empirical parameter *d *are shown in Table 4. This was impossible for the RNA atoms around unpaired pyrimidine bases, because of the paucity of data.

While the interactions with other RNA molecules of the same asymmetric unit were limited to stacking contacts, clusters of crystal symmetry related atoms were found close to the N1 nitrogen atom of guanine and close to the atoms N3 and O2 of cytosine. These clusters are compatible with the presence of hydration sites. It is thus possible that absence of water molecules close to these three RNA atoms is due, at least in part, to the fact that the space close to these three RNA atoms is occupied, in the crystal structures, by symmetry related molecules. This hypothesis is also supported by the observation that several clusters of atoms of proteins, ligands, and ions are found around guanines and adenines, mimicking their solvatation. Surprisingly, this was not observed for the smaller pyrimidines.

### Atomic displacement parameter

The atomic displacement parameter, or B-factor, is determined by molecular plasticity/rigidity and conformational disorder [31-35]. We have carried out a comparative analysis of B-factors of different RNA and water atoms in order to estimate the degree of rigidity of the water molecules in the clusters around the bases of unpaired RNA nucleotides. First, B-factors were normalized to zero mean and unit variance [31-35] with the following equation:

(5) $$B^{N}=\frac{B-B_{a}}{B_{\sigma }}$$

where *B *is B-factor of the RNA or water atom, *B_a _*is the average B-factor and *B_σ _*is the standard deviation computed over all the atoms. Such normalization is necessary for a number of reasons: the B-factors may be influenced by numerous computational details and their values and variability might not reflect genuine physical features; moreover, although most of the crystal structures examined in the present paper were determined in the 100-120 Kelvin range of temperature, some were determined at a lower or higher temperature (even at room temperature in few cases).

Table 5 shows the average *B^N ^*values, with their standard deviations, for the RNA base atoms of paired and unpaired nucleotides and for the oxygen atoms of the water molecules that were grouped into the clusters described above and for the oxygen atoms of the other water molecules that could not be grouped into discrete clusters.

**Table 5:** Atomic displacement parameter

Base	Atoms of paired bases	Atoms of unpaired bases	Oxygen atoms of water molecules outside clusters	Oxygen atoms of water molecules inside clusters
Adenine	0.393 ± 0.005	0.798 ± 0.010	1.09 ± 0.04	0.95 ± 0.13 (W1) 1.63 ± 0.15 (W2) 1.21 ± 0.11 (W3) 1.96 ± 0.16 (W4) 1.68 ± 0.15 (W5)

Guanine	0.342 ± 0.003	0.827 ± 0.014	1.20 ± 0.05	1.31 ± 0.19 (W1) 1.39 ± 0.18 (W2) 1.59 ± 0.28 (W3) 1.73 ± 0.17 (W4) 0.71 ± 0.12 (W5) 1.36 ± 0.28 (W6) 1.61 ± 0.15 (W7)

Uracil	0.350 ± 0.005	0.967 ± 0.018	1.07 ± 0.05	0.82 ± 0.13 (W1) 0.91 ± 0.22 (W2) 0.96 ± 0.26 (W3) 1.41 ± 0.41 (W4)

Cytosine	0.384 ± 0.004	0.962 ± 0.020	0.85 ± 0.06	0.70 ± 0.14 (W1) 0.80 ± 0.26 (W2) 1.00 ± 0.21 (W3)

Average normalized B-factors (*B^N^*, equation 7) with their standard deviations, computed for different types of atoms: base atoms belonging to paired nucleotides; base atoms belonging to unpaired nucleotides; oxygen atoms of water molecules that do not belong to clusters; oxygen atoms of water molecules that reside into the clusters described in Table 2.

The *B^N ^*values of the unpaired nucleotides are more than the double than the *B^N ^*value of the nucleotides that form base-pairs. Unpaired nucleotides of pyrimidines, that contain only one heterocyclic ring, are less rigid than purines, which have two rings. On the contrary, there is less difference in the *B^N ^*values of purines and pyrimidines that form base-pairs.

Water molecules that could not be grouped into discrete clusters have relatively large *B^N ^*values, are slightly smaller around cytosine. Water molecules that cluster into the hydration sites described above have *B^N ^*values that are quite variable. However, on average, they resemble the *B^N ^*values of the other water molecules and of the unpaired base atoms. This indicates clearly that solvent dynamics are correlated with base mobility.

## Conclusions

Hydration sites around unpaired RNA bases were found. Many of them match the hydration sites found in the major and minor grooves of RNA and DNA double helices. On the contrary, they do not replicate the atom positions of complementary bases in the Watson-Crick pairs. The Watson-Crick edge of the unpaired bases interacts more frequently with proteins, ligands, and ions than with water molecules. The hydration site positions defined in the present study can be used to analyze RNA structure and function, in drug design and modeling interactions, and in the last stages of crystallographic refinements.

## Methods

### Data selection

1393 crystal structures, containing 2930 sequences, were taken on the 20 December 2010 from the Protein Data Bank [36]. Highly identical and redundant sequences were removed with CLEANUP at 90% sequence identity [37]. The same [38] or higher (95%) [39] levels of identity are used for the preparation of the non-redundant datasets in other studies of RNA structures. Structures with crystallographic resolution worse than 2.0 Å were removed and unpaired nucleotides were indentified with the 3DNA software package using the default setting of the base pairing parameters [40]. List of the PDB files chosen for the statistical analysis is represented in the Additional file 5. Pertinent details on the data examined in the present paper are shown in Table 1. Although not all the water molecules deposited in the Protein Data Bank might be genuine water molecules (some of them might be in the reality mono-atomic ions), we accepted the data as they are. On the one hand, we considered only high resolution crystal structures, where mistakes are less probable. On the other hand, methods for identifying mono-atomic ions (see for example [41,42]), were calibrated on protein crystals, and are not necessarily reliable on RNA crystals. It appears that there are somewhat limited amounts of data available. For example, only 405 high-resolution adenine bases are available, out of the 18, 756 adenine bases presented in the crystal structures examined in the present paper. This remarkable reduction of data is principally due to the fact that a small number of ribosomal structures provide large numbers of low resolution data.

Water molecules within 5.0 Å from the base atoms were considered. A more strict threshold value (3.2 Å) was used in the statistical study of DNA hydration carried out by Schneider and coworkers [16]. A less stringent value (3.4 Å) was then used by Schneider and Berman [17] in a further study and an even larger value (4.0 Å) was used by Auffinger and Westhof in an analysis of the first hydration shell around the RNA base pairs [18]. In the present study, a large cut-off distance of 5.0 Å was chosen to avoid strict boundary conditions. Interestingly, this allows the detection of some solvation sites of the second hydration shell, which are bound to the RNA bases via a hydration site of the first solvation shell. However, it should be observed that the hydration sites of the second hydration shell might not correctly describe the hydration of the unpaired RNA bases in solution, since they can be strongly influenced by the crystal packing interactions.

Ideal stereochemistries of bases were taken from [43]. Each base examined in the present paper was superposed to its ideal reference model, with the Kabsch algorithm [44]. By applying the rotations and translation to the water oxygen atoms, all water molecules surrounding the bases were placed into the same reference frame.

The analyses reported in the present paper disregarded the sequence environment of the bases and their secondary structure because of the paucity of the data.

### Cluster detection

Cluster analysis is widely used in different scientific areas for unsupervised pattern recognition [45,46]. In the present study, we have applied Monte Carlo simulations for detecting spatial point clusters [47]. Clusters of water molecules are detected by large numbers of individual water molecules within a sphere, while the position and dimension of the sphere are systematically varied.

First, the boundaries of the space around the unpaired RNA bases were determined. The minimal and maximal distances between the water oxygen atoms and the centers of mass of the bases were found to be equal to 2.5 and 8.0 Å for purines, and 2.3 and 7.8 Å for pyrimidines.

Moreover, the critical number of points within the sphere is given by the empirical function *L*

(6) L(r)=d⋅r,

where *r *is radius of the sphere and *d *is a parameter defined by using the following procedure.

*N *random points are selected in the space populated by water molecules around the base (*N *= 719 for adenine, 474 for guanine, 250 for cytosine, and 373 for uracil; see Table 1). The number *n *of random points contained in a randomly positioned sphere of radius equal to 1.3 Å (about half the distance of adjacent water molecules in the solid state - 2.75 Å) is then inserted in the expression

(7) σ=d⋅1.3-n,

This procedure is iterated 999 times and the value of *d *was selected empirically in such a way that σ assumes values smaller than 0 in 50 cases and bigger than 0 in the remaining 949 cases. This means that the probability to find more than *L(r = 1.3) *points in the sphere of radius equal to 1.3 Å is 0.05 if the points are distributed randomly. The *d *values, which depend on *N*, are equal to 24, 16, 13, and 11 for adenine, guanine, uracil, and cytosine, respectively.

The space around the unpaired base occupied by water molecules was scanned using a discrete grid, defined in a spherical coordinate system, with the origin on the center of mass of the base, with radius increments of 1.0 Å and increments of the two spherical angles equal to 3 degrees. Spheres were centered at the grid intersections, with a variable radius that increases from 0.2 to 0.8 Å with steps of 0.1 Å. In the method described in [47] a broader lattice is reconstructed when the radius increases. In our algorithm, a constant fine-grained lattice is used during the calculations that results in some overlapping of the clusters, which was removed with the following procedure. Let *X *= {*x_i_*} to be the oxygen atoms of a cluster and let *Y *= {y_*i*_} to be the atoms of another cluster: any element of *Y *is moved to *X *if it is within 0.5 Å from any of the elements of *X *and the procedure is iterated until convergence.

## Authors' contributions

SK developed the computational tools, carried out the calculations, and drafted the manuscript. OC initiated and supervised the project. SK and OC read and approved the manuscript.

## Supplementary Material

Additional file 1 **Hydration sites around the guanine unpaired base**. PDB file that contains the most populated nucleotide conformations and the clusters of water molecules around guanine unpaired RNA base corresponding to the hydration sites. Click here for file

Additional file 2 **Hydration sites around the adenine unpaired base**. PDB file that contains the most populated nucleotide conformations and the clusters of water molecules around adenine unpaired RNA base corresponding to the hydration sites. Click here for file

Additional file 3 **Hydration sites around the cytosine unpaired base**. PDB file that contains the most populated nucleotide conformations and the clusters of water molecules around cytosine unpaired RNA base corresponding to the hydration sites. Click here for file

Additional file 4 **Hydration sites around the uracil unpaired base**. PDB file that contains the most populated nucleotide conformations and the clusters of water molecules around uracil unpaired RNA base corresponding to the hydration sites. Click here for file

Additional file 5 **List of RNA structures**. List of the PDB files used in the present study. Click here for file
