# Supplementary material for: Hydration sites of unpaired RNA bases: a statistical analysis of the PDB structures
Source: BMC Struct Biol. 2011 Oct 19;11:41. doi: 10.1186/1472-6807-11-41 (PMC3206426; doi:10.1186/1472-6807-11-41)
Supplement: Additional file 5 — List of RNA structures. List of the PDB files used in the present study. [file 1472-6807-11-41-S5.PDF]

N	ID	chain	Resolution, A	Temperature, Kelvin
1	2A43	A	1.34	110
2	1UVL	F	2.00	150
3	3BNQ	A	2.00	100
4	1B2M	D	2.00	287
5	2XNR	C	1.60	100
6	1DUL	B	1.80	100
7	1UVL	D	2.00	150
8	1L2X	A	1.25	100
9	3DS7	A	1.85	100
10	1B2M	E	2.00	287
11	1B2M	C	2.00	287
12	1UTF	8	1.90	120
13	1UTV	5	1.90	120
14	1U8D	A	1.95	100
15	3CJZ	B	1.80	120
16	2X1F	B	1.60	100
17	1NUJ	H	1.80	100
18	3GPQ	F	2.00	100
19	1UTF	3	1.90	120
20	1ZEV	B	1.58	100
21	1UTV	4	1.90	120
22	1UVM	F	2.00	150
23	2ZY6	A	1.75	100
24	3BOY	D	1.70	100
25	1UTV	3	1.90	120
26	2PXE	B	2.00	113
27	3K62	B	1.90	100
28	2PXD	B	2.00	113
29	1UTF	4	1.90	120
30	406D	E	1.80	110
31	1SDS	D	1.80	100
32	1UTF	0	1.90	120
33	2OE5	A	1.51	100
34	1IK5	B	1.80	140
35	1UTF	6	1.90	120
36	1UTV	9	1.90	120
37	1UTV	7	1.90	120
38	1T0E	C	1.70	103
39	2OE5	B	1.51	100
40	3K64	B	2.00	100
41	2B3J	E	2.00	100
42	1UTV	0	1.90	120
43	3LA5	A	1.70	180
44	2PXB	B	2.00	113
45	1UTF	1	1.90	120
46	1UTF	5	1.90	120
47	2R1S	B	1.40	80
48	2F8K	B	2.00	100
49	3CGP	A	1.57	100
50	3DD2	B	1.90	98
51	1F27	A	1.30	100
52	1T0E	A	1.70	103
53	1UTF	7	1.90	120
54	1R9F	B	1.85	100
55	1C9S	W	1.90	120
56	1UTV	1	1.90	120
57	3GPQ	E	2.00	100
58	1H2C	R	1.60	100
59	2R8S	R	1.95	100
60	1UTF	Z	1.90	120
61	2Q66	X	1.80	100
62	1R9F	C	1.85	100
63	1UTF	2	1.90	120

64	1UTV	8	1.90	120
65	1UTV	Z	1.90	120
66	1J1U	B	1.95	100
67	3CGP	B	1.57	100
68	1UVM	D	2.00	150
69	1UVM	E	2.00	150
70	2ANR	B	1.94	100
71	1UTV	6	1.90	120
72	2ZUE	B	2.00	100
73	1UVL	B	2.00	150
74	2PXF	B	2.00	113
75	1UTV	2	1.90	120
76	1GTF	W	1.75	120
77	1UTF	9	1.90	120
78	1CSL	B	1.60	100
79	1KFO	A	1.60	NULL*
80	1URN	P	1.92	NULL*
81	2PXV	B	2.00	113
82	1K8W	B	1.85	100

*NULL - The temperature is not not provided in the PDB file.
